# Metasurface for programmable quantum algorithms with classical and quantum light

**DOI:** 10.1515/nanoph-2023-0844

**Published:** 2024-02-21

**Authors:** Randy Stefan Tanuwijaya, Hong Liang, Jiawei Xi, Wai Chun Wong, Tsz Kit Yung, Wing Yim Tam, Jensen Li

**Affiliations:** Department of Physics, The Hong Kong University of Science and Technology, Clear Water Bay, Kowloon, Hong Kong, P.R. China

**Keywords:** programmable metasurface, quantum information, quantum optics, quantum algorithms

## Abstract

Metasurfaces have recently opened up applications in the quantum regime, including quantum tomography and the generation of quantum entangled states. With their capability to store a vast amount of information by utilizing the various geometric degrees of freedom of nanostructures, metasurfaces are expected to be useful for processing quantum information. Here, we propose and experimentally demonstrate a programmable metasurface capable of performing quantum algorithms using both classical and quantum light with single photons. Our approach encodes multiple programmable quantum algorithms and operations, such as Grover’s search algorithm and the quantum Fourier transform, onto the same metalens array on a metasurface. A spatial light modulator selectively excites different sets of metalenses to carry out the quantum algorithms, while the interference patterns captured by a single-photon camera are used to extract information about the output state at the selected output directions. Our programmable quantum metasurface approach holds promising potential as a cost-effective means of miniaturizing components for quantum computing and information processing.

## 1 Introduction

While superconducting circuits and trapped ions are two promising physical technologies for implementing quantum computing, photonic implementation remains a versatile platform [1], [2], [3]. Photonic implementation not only allows for the investigation of fundamental questions in quantum mechanics [4], [5], [6] but also provides a practical way to demonstrate the advantages of quantum algorithms in solving problems that are significantly slower on classical computers [7], [8], [9]. In fact, classical optics already possesses key elements of quantum computing, such as superposition, interference, and to a large extent, non-separable states analogous to entanglement without quantum non-locality [10], [11]. It becomes possible to construct non-separable states and perform quantum algorithms by utilizing the different internal degrees of freedom of single photons, even using classical light [12], [13]. This approach has already been adopted in real applications, such as quantum key distributions to increase key rates [14].

Tremendous efforts have been devoted to simulating various quantum algorithms using classical light as an easily accessible testbed. For instance, quantum algorithms involving up to two-qubit operations can be constructed using common optical components, such as beam splitters, polarization beam splitters, lenses, and Dove prisms, by leveraging the two paths and polarization degrees of freedom [12], [13]. Recent experiments have explored the use of vortex beams generated or manipulated by a spatial light modulator (SLM) to achieve quantum algorithms with up to 4 qubits, utilizing 16 orbital angular momenta (OAM) [15], [16]. Additionally, more efficient and programmable utilization of the SLM has demonstrated the implementation of the Deutsch–Jozsa algorithm with an effective complexity of 20 qubits [17], [18]. These principles can also be applied at the single-photon level when using a quantum source, where practical demonstrations often employ the heralding technique to enhance detection purity and signal-to-noise ratio [18], [19], [20]. Moreover, by working with photon counting in the quantum regime, it becomes possible to investigate two-photon physics within the same linear optical system. It can further expand the ways to construct qubits and quantum algorithms, including boson sampling for evaluating the permanents of matrices [21] and entangled cluster states for one-way quantum computation [22].

On the other hand, metasurfaces, which consist of a single layer of nanostructures, have proven to be highly useful in multiplexing or demultiplexing optical information. They have been applied successfully in generating vortex beams or holograms with fine resolution [23], [24], [25], [26], [27], [28]. As we move towards the quantum regime, we have been witnessing early demonstrations of metasurfaces in quantum tomography, the generation of high-dimensional entanglement, quantum imaging, and the manipulation of two-photon interference [29], [30], [31], [32], [33], [34], [35]. Furthermore, due to their capability to store a significant amount of information regarding light–matter interaction, metasurfaces offer the potential for processing quantum optical information and implementing quantum algorithms. Some studies have proposed using metamaterials to simulate quantum algorithms in the classical domain [36], [37], [38] and to solve differential equations [39], [40].

In this work, we have developed a platform with a tailor-made geometric-phase metalens array on a metasurface to execute programmable quantum algorithms, enabling the implementation of unitary transformations in a single step. By employing a spatial light modulator (SLM) and a single-photon camera for the generation and detection of input and output states, we have performed programmable quantum algorithms and operations in the optical domain. In this case, our work demonstrates programmability on a static metasurface by using a spatial light modulator (SLM) to selectively illuminate different sets of metalenses and a single photon camera to selectively observe at different exit directions. Similar approaches using static metasurfaces have been used in generating dynamic holograms by modulating incident polarization, orbital angular momentum, and spatial profile [41], [42], [43]. It allows us to implement Grover’s search (GS) and the quantum Fourier transform (QFT). GS algorithm is a quantum search algorithm to find a unique marked element in an unstructured database that offer a quadratic speedup over the classical search algorithm [44]. We have chosen the GS algorithm for its practical significance, as fast searching facilitates solving difficult computational problems for future extension [22]. Likewise, QFT, a quantum equivalent of the discrete Fourier transform, serves as a fundamental algorithmic primitive that enables the implementation of several other quantum algorithms, with the most notable being Shor’s algorithm [45]. To substantiate our methodology, we conducted comprehensive simulations and experiments employing both classical and quantum light sources. The inherent programmability of our strategy heralds a new era in the miniaturization of quantum optical circuits, presenting an economical and integrated avenue for quantum information processing.

## 2 Results and discussion

### 2.1 Computing scheme using metalens array for programmable quantum algorithms and operations

**Figure 1:**
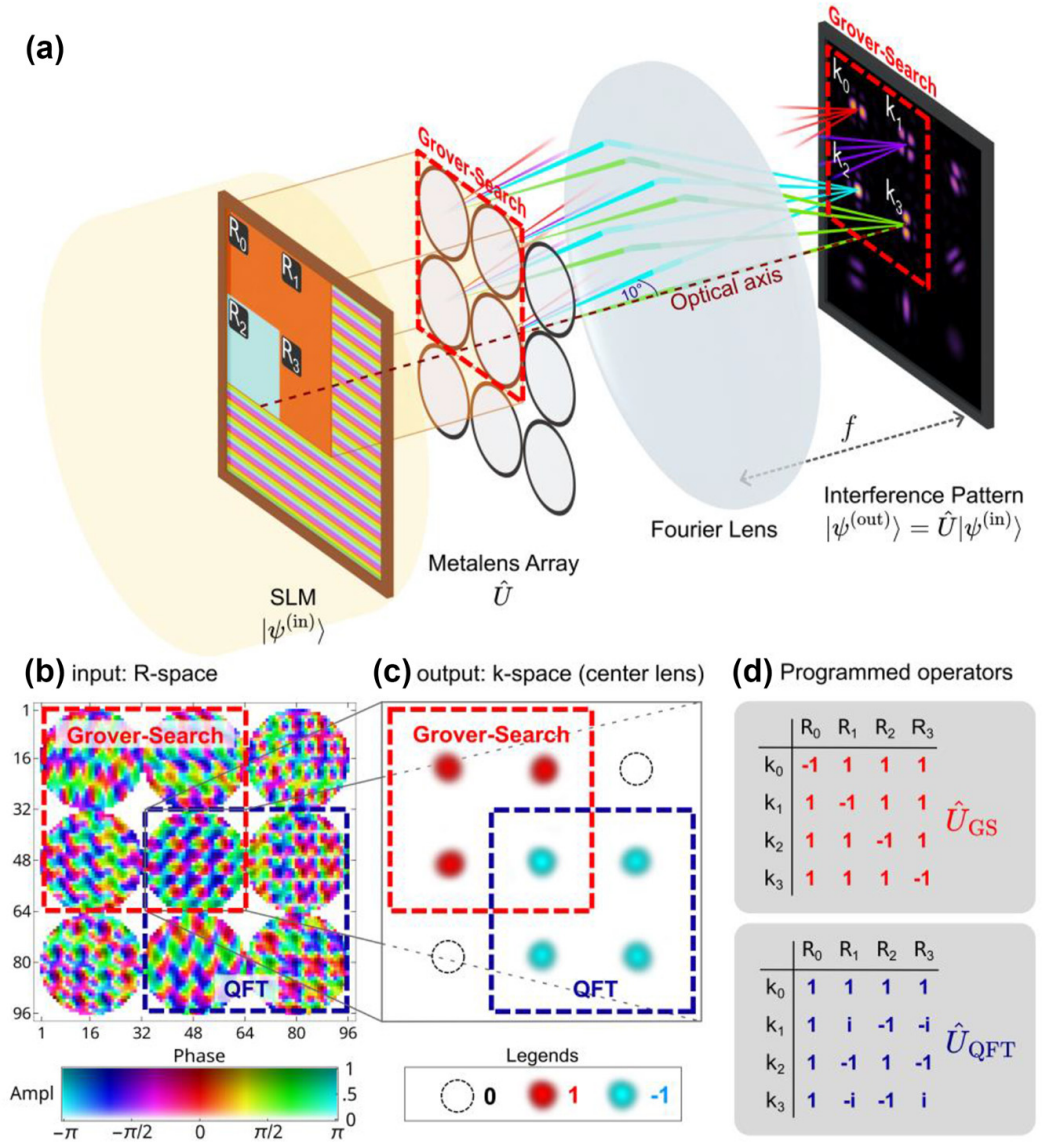
Computing metasurface for programmable quantum algorithms. (a) Overall schematic of the computing metasurface for implementing two-qubit unitary quantum operations. A spatial light modulator (SLM) selectively excites the metalens array and prepares the two-qubit input state $\left|\psi^{\left(\text{in}\right)}\right\rangle$ by modulating the complex incident E-field for each selected metalens at **R**_0_ to **R**_3_. Each metalens diffracts the beam into multiple output directions, with amplitude and phase modulated by the *U*-matrix. The interference patterns of the diffracted beams in the same direction are projected onto the Fourier plane, where the intensity profile around **k**_0_ to **k**_3_ yields the output state $\left|\psi^{\left(\text{out}\right)}\right\rangle =\hat{U}\left|\psi^{\left(\text{in}\right)}\right\rangle$ . (b) Complex transmission amplitude and phase profile of the metalens array, encoding both Grover’s Search (GS) and Quantum Fourier Transform (QFT). (c) Far-field intensity profile when only the center lens is excited to implement a column of the unitary *U* matrix. The color shows different amplitude and phase modulation for different output directions (all of the same wavelength). Programmability is achieved by selecting the metalens excitation (**R**_0_ to **R**_3_) and the projected interference patterns (**k**_0_ to **k**_3_). For example, selecting the red square in (b) and (c) enables GS, while selecting the blue square enables QFT. (d) The corresponding matrices for the two programmed quantum operators, ${\hat{U}}_{\text{GS}}$ and ${\hat{U}}_{\text{QFT}}$ (normalization factor omitted for brevity).

Our goal is to implement a specific quantum algorithm for *n*-qubits, which can be summarized as a transformation: $\left|j\right\rangle \to \sum_{i=0}^{2^{n}-1}U_{ij}\left|i\right\rangle$ , where *U* is a unitary matrix, using a metasurface. The ‘ket’ notation represents the n-qubit basis for either the input or output state, with the integer *j* or *i* ranging from 0 to 2^*n*−1^. Our metasurface approach is shown in Figure 1(a). A spatial light modulator (SLM) is utilized to prepare the input state $\sum_{j=0}^{2^{n}-1}\psi_{j}^{\left(\text{in}\right)}\left|j\right\rangle$ , where input basis $\left\{\left|j\right\rangle \right\}$ represents a uniform electric-field normally incident on the lens located at **R**_*j*_, and $\psi_{j}^{\left(\text{in}\right)}$ represents the complex field coefficient of the corresponding basis. The SLM operates in vertical polarization, which is subsequently converted to left-circular polarization (LCP) before illuminating the metasurface. Our metasurface comprises 9 metalenses, of which four are employed to perform a specific 2-qubit (*n* = 2) unitary operation. Each metalens at **R**_*j*_ diffracts the input mode $\left|j\right\rangle$ into a discrete set of output modes $\left\{\left|i\right\rangle \right\}$ , with the corresponding exit direction **k**_*i*_ and designed complex modulation *U*_*ij*_. The basis of the output state $\left\{\left|i\right\rangle \right\}$ corresponds to an exit beam centered at **k**_*i*_ on the far-field of the metasurface. The different colors, physically all at the same wavelength, illustrate different output directions; for instance, a green beam corresponds to the normal exit direction, while a cyan beam corresponds to a 10° deflection to the left. In our case, each metalens converts LCP to right-circular polarization (RCP) using a custom complex transmission amplitude and phase profile given by the expression:

(1) $$\begin{array}{c} t_{j}\left(\rho \right)=\overset{2^{n}-1}{\underset{i=0}{\sum }}U_{ij}\exp\left(\text{i}k_{i}\cdot \rho \right), \end{array}$$

where **ρ** denotes the transverse position measured from the center of the entire metasurface. Consequently, light from different metalenses with the same output direction interferes in the far-field regime, resulting in the output mode $\left|i\right\rangle$ in RCP. By fitting the interference pattern captured by a single-photon camera, we can obtain the output field amplitude $\left|\psi_{i}^{\left(\text{out}\right)}\right|$ . The entire unitary operation can be summarized as $\psi_{i}^{\left(\text{out}\right)}=\sum_{j=0}^{2^{n}-1}U_{ij}\psi_{j}^{\left(in\right)}$ in terms of the complex field coefficient.

Our scheme incorporates programmability using a static metasurface with shared resources for implementing different quantum algorithm choices through selective illumination. The complex input field coefficient $\psi_{j}^{\left(\text{in}\right)}$ on a specific metalens at **R**_*j*_ is prepared by the corresponding region of a (phase) SLM at normal incidence. A certain fraction of these pixels is deactivated by introducing a phase gradient, diverting the light at an angle that does not fall on the lens array. This process can set $\psi_{j}^{\left(\text{in}\right)}$ with both amplitude and phase [35]. The metalens then implements the *j*th column of the unitary matrix *U* through a transmission profile at each lens. Figure 1(b) illustrates a typical transmission profile (amplitude and phase in a color map) of the metasurface, comprising a total of 9 metalenses. Our metalens array is programmed with two different quantum operations: Grover’s search (GS) or quantum Fourier transform (QFT), achieved by illuminating a 2-qubit input state on the 4 upper-left (dashed red square) or lower-right lenses (dashed blue square) in Figure 1(b), referred to as R-space, using the SLM. The lens labels **R**_0_ to **R**_3_ for the GS are indicated in Figure 1(a), while the same lens labels are defined in a similar row-major order for the lenses used in QFT. On the other hand, the four output directions **k**_0_ to **k**_3_ for the GS or QFT are shown in Figure 1(c), referred to as k-space, again using dashed red square or blue square. For convenience, we have adopted the same label order as the convention used for R-space, but other choices are possible as long as 4 out of the 9 directions are selected as outputs in defining the *U* matrix. The corresponding unitary matrices of GS (*U*_GS_) and QFT (*U*_QFT_) are shown in Figure 1(d) (normalization factor omitted). Taking the center lens, **R**_3_, as an example, it generates {1, 1, 1, −1} at its 4 output directions for *U*_GS_, or {−1, −1, −1, −1} (for the same lens now labeled as **R**_0_) at its 4 output directions for *U*_QFT_ (a global *π* phase is added to the entire matrix without altering unitarity). Thus, **R**_3_ → **k**_3_for *U*_GS_ and **R**_0_ → **k**_0_ for *U*_QFT_ both implement the same matrix element −1, indicating that this resource is shared between the two operators. The matrix elements, with one of them shared and implemented by the center lens, are shown in Figure 1(c). The sharing of resources allows for a more compact implementation of the metalens array given limited resources.

### 2.2 Geometric-phase metasurface and experimental setup

**Figure 2:**
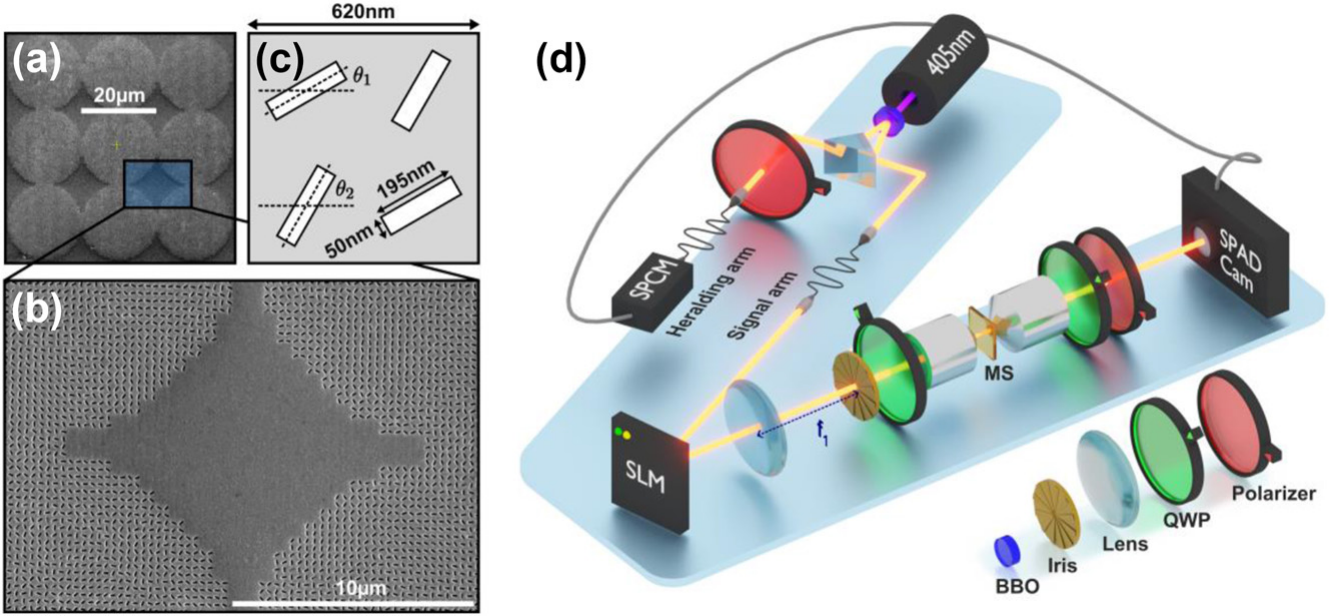
Geometric-phase metalens array and heralding photon experimental setup. (a) SEM image of the metasurface designed for operational wavelength *λ* = 810 nm. (b) Enlarged SEM view of the metalens array showcasing the quality of the metalens. (c) Unit cell for controlling the amplitude and phase response, composed of two pairs of rotated nano-air-slots (50 nm × 195 nm) on a silver film (50 nm thick) coated glass substrate, with variable orientations *θ*_1_ and *θ*_2_. (d) Experimental setup using a heralding photon source for imaging the far-field interference patterns resulting from the quantum operation.

Figure 2(a) showcases the Scanning Electron Microscope (SEM) image of the metasurface, with a close-up view provided in Figure 2(b). The design consists of nine metalenses, each comprising 32-unit cells along the diameter (approximately 20 μm in diameter). Each metalens is composed of nano-slots with variable orientations. In particular, each unit cell, with a periodicity of *p* = 620 nm, contains two pairs of rotated nanoslots (195 nm long, 50 nm wide) with rotation angles *θ*_1_ and *θ*_2_, as depicted in Figure 2(c). The chosen periodicity is less than the operational wavelength of *λ* = 810 nm to suppress higher-order diffraction. Regarding the two pairs of rotating slots within a unit cell, we focus on achieving cross-circular polarization (CP) from LCP to RCP in our scheme, contributing to a transmission with an amplitude proportional to $\left|\cos\left(\theta_{1}-\theta_{2}\right)\right|$ and an overall geometric phase of *θ*_1_ + *θ*_2_ [46]. By selecting appropriate values of *θ*_1_ and *θ*_2_ at each unit cell within the metalens at **R**_*j*_, the resultant complex transmission amplitude profile is designed to match the specifications outlined in Eq. (1), where now the *U* -matrix represents the whole 9 × 9 transfer matrix obtained by combining all the unitary matrices of the programmed quantum operators. We note that the amplitude control accompanies a power loss in transmission. Here, *i* ranges from 0 to 8, corresponding to the 9 output directions from the same lens at **R**_*j*_. Moreover, for the entire (9 lenses) × (9 directions) matrix, there are unused matrix elements where we can embed more operations if needed (for further discussion, see Supplementary Materials). In addition, an overall normalization constant and a quadratic focusing phase profile are actually added to the metasurface transmission profile, integrating the schematic Fourier lens shown in Figure 1(a) onto the metalens array for the convenience of characterization (see Supplementary Materials). The design of the metalens array, incorporating the nanoslots with the orientation profile, is fabricated on silver-coated glass (50 nm coating) using the focused ion beam (FIB) technique.

The experimental setup, incorporating an entangled quantum light source, is illustrated in Figure 2(d). A 405 nm laser (CrystaLaser DL-405-400) pumps a type-II β-barium borate (BBO) crystal to generate two photons at a wavelength of 810 nm in a polarization-entangled state of $1/\sqrt{2}\left(\left|HV\right\rangle +\left|VH\right\rangle \right)$ , with one photon sent to the heralding arm and the other to the signal arm. A polarizer on the heralding arm selectively filters horizontally (H) polarized photons, which are then detected by a Single Photon Counting Module (SPCM) (Excelitas-SPCM-800-14-FC), triggering the Single Photon Avalanche Diode (SPAD) camera (Pi Imaging SPAD 512S) located at the end of the signal arm. The SPAD camera operates in gated mode with a 16-ns coincidence window. The heralding process only triggers imaging for those photons with vertical (V) polarization that arrive at the Spatial Light Modulator (SLM) (Holoeye Pluto 2.1) in the signal arm. The SLM generates the input state and selectively excites the metalens. As mentioned earlier, to control the amplitude from the phase-SLM, some light is diverted and blocked by an iris positioned at the focal plane of a lens with *f*_1_ = 40 cm. A pair of orthogonal Quarter Wave Plates (QWP) is placed before and after the metasurface to convert the linearly polarized incident beam to circular polarization (CP) and vice versa. The first QWP, with a fast axis oriented at the diagonal, converts V-polarized light to LCP. Upon interacting with the metasurface, the photons diffract into different output directions. The second QWP, with a fast axis oriented at the anti-diagonal, and an H-polarizer are used to convert the polarization back to linear polarization that is orthogonal to the input light in the signal arm. Consequently, interference patterns corresponding to different output directions are formed at the focal plane of the metalens array and captured by the SPAD camera. Extended exposure time (a total of 20,000 frames with each frame lasting 0.3s) is utilized for data acquisition due to the extremely low photon count from the quantum source, and the use of the heralding technique is essential to increase the signal-to-noise ratio for detection in the case of quantum light source.

### 2.3 Experimental characterization

We first examine the operations of the metasurface using classical light. In this case, we replace the part before the SLM in Figure 2(d) with an 810 nm laser (OBIS LX 808 nm 150 mW Laser). The utilization of classical light for excitation effectively enhances the signal, resulting in shorter acquisition times (5–10 ms). It also leads to higher resolution in the interference patterns formed at the focal plane of the metalens array because the higher power can be distributed among more pixels on the single-photon camera. These investigations facilitate the more challenging task of extracting information from the interference patterns in the subsequent stage with a quantum light source.

**Figure 3:**
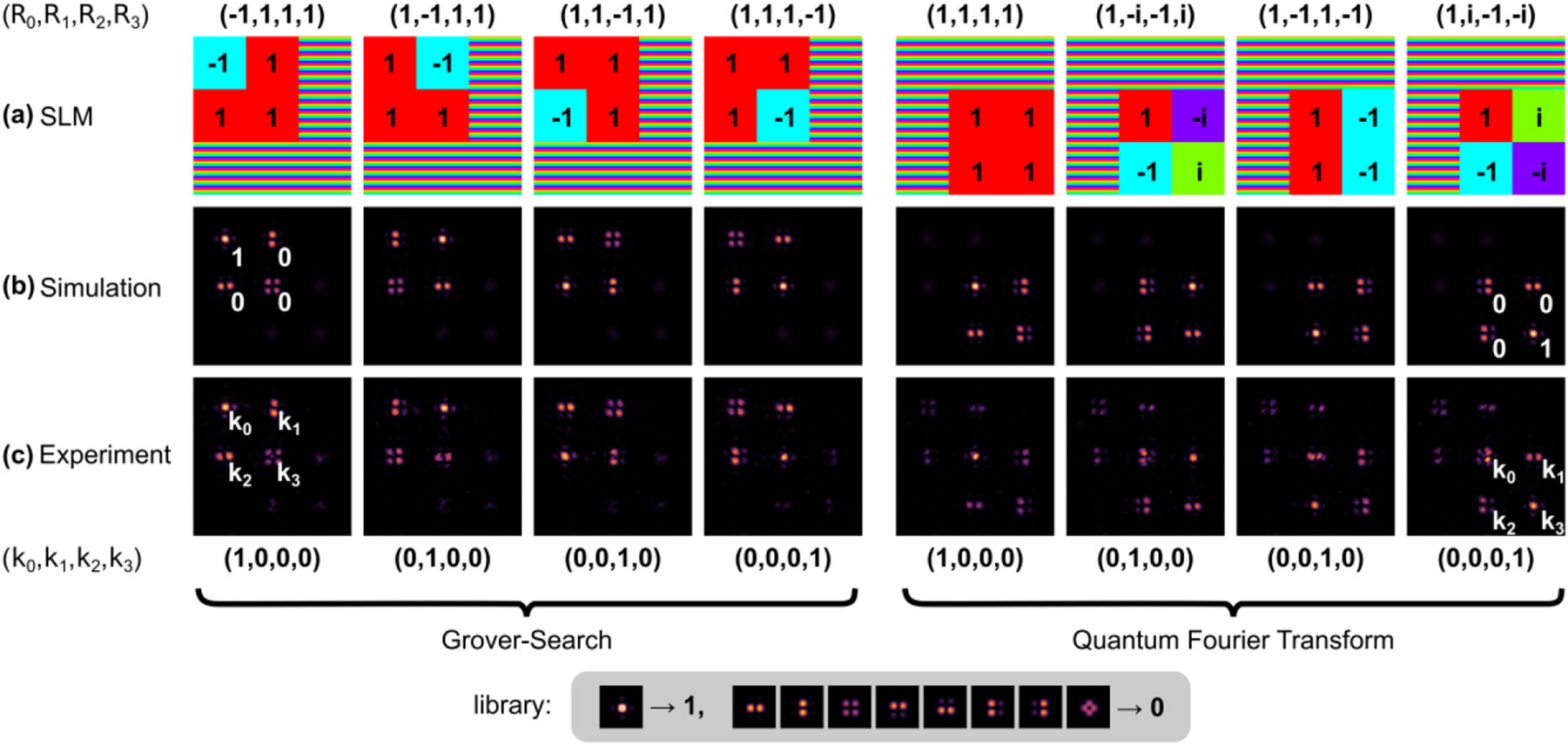
Simulation and experimental results using a classical light source for the Grover-Search (left) and QFT (right) operations. (a) SLM phase profile for each input state for the two operations: four input states for each operation listed at the top of each column. Color code: red for 1, cyan for −1, green for *i*, and purple for −*i*, shaded regions (phase gradient) are effectively inactive. (b) Numerical simulation of the far-field interference pattern. (c) Experimental result using a classical light source. The input and output states are labeled at the top and bottom of each column. The library for decoding the interference pattern into the output state for the representative cases of values 1 and 0 is listed in the bottom inset, where constructive interference (1) exhibits a bright center node and destructive interference (0) exhibits a dark center node.

Figure 3 displays the simulation and experimental results obtained using a classical light source for two operations: GS (left 4 columns) and QFT (right 4 columns). For each operation, we test it using four different input states, which are labeled at the top of each corresponding result column. Let us begin with the first column, which corresponds to the input state $\left(-1,1,1,1\right)$ for the GS algorithm. In the current case, the GS algorithm essentially picks the basis with *π* phase shift (a unique marked element in the database) and output the state with zero components except the corresponding basis with value 1: (1, 0, 0, 0), with ideally unit probability on the corresponding basis using the metalens array to implement the unitary matrix *U*_GS_ (as shown in the matrix multiplication in Figure 1(d)). The input state is initially encoded as the phase profile on the SLM (Figure 3(a)) using the four upper-left lenses in a row-major manner: a region marked as −1 has a constant SLM phase of π (cyan color), while a region marked as 1 has a constant SLM phase of 0 (red color). Only the corresponding metalenses are selectively excited to implement the GS algorithm, while the other regions display a phase gradient to deflect the light away from the metalens array. In the far field (practically at focal plane of the metalens), the interference patterns are formed at the output directions, as shown in the numerical simulation (by using angular spectrum method to propagate the complex field to the focal plane of the metalens) in Figure 3(b) and the experimental results captured by the single-photon camera in Figure 3(c). The interference patterns (intensity) for the two results closely resemble each other and represent the output state in the upper left region, also indexed in a row-major manner. The intensity at direction **k**_*i*_, proportional to ${\left|\psi_{i}^{\left(\text{out}\right)}\right|}^{2}$ , can then be determined from the center of each interference pattern. It is worth noting that only the upper left 4 directions exhibit notable interference patterns (as seen Figure 1(c)). To achieve higher accuracy, we extract the intensity using the entire interference pattern. Some representative cases from the library are shown at the bottom of Figure 3, illustrating the different possible patterns for value 0 (fully destructive, resulting in dark centers) and 1 (fully constructive, resulting in a bright center). By comparing the interference patterns with the library, we find that the output state agrees with the expected result $\left(1,0,0,0\right)$ for the GS algorithm. In the meantime, for the sake of convenience of our discussion, we discretize the output state to be either fully destructive or fully constructive interference. More details are given in Supplementary Materials regarding the decoding process for the full spectrum of intensity values other than 0 and 1, which will be used in the next step.

For completeness, the results for the other three different input states with *π* phase change at the corresponding input basis are shown in the second to fourth columns. The simulation and experiment results exhibit good agreement, while the decoded output states are shown below the experimental results. To test the quantum Fourier transform (QFT) using the same technique, we select the input states to be (1, 1, 1, 1), (1, −*i*, −1, *i*), (1, −1, 1, −1) and (1, *i*, − 1, − *i*) in order to detect the different “frequencies” in the state. The SLM sets the corresponding phases, as shown in the first row of Figure 3(a). The output states correspond to the columns of the 4 × 4 identity matrix, corresponding to the four lower-right output directions exhibiting significant interference patterns. The simulation and experimental interference patterns are in agreement and can be decoded (in a row-major manner) to yield the expected output states.

With the previous experience gained from using classical light to confirm the metasurface’s functionality, we now shift our focus to discussing the results obtained with a quantum light source. While we expect consistent results by using the two different types of sources, the utilization of a quantum light source highlights a key aspect: a single-photon can simultaneously interact with all nine metalenses (in a similar spirit to a double Young slit experiment), which shows the metasurface’s potential as a low-power platform for implementing quantum algorithm. It is important to note that due to the probabilistic nature of single-photon detection, obtaining an accurate output state necessitates sampling numerous measurements of single photons. This aligns with the conventional approach of implementing quantum algorithms through a quantum circuit, which involves executing the algorithm multiple times. Repeated measurement plays a vital role in some algorithms, such as in GS to reduce the risk of false positives and in QFT to achieve a precise probability distribution [7], [22].

**Figure 4:**
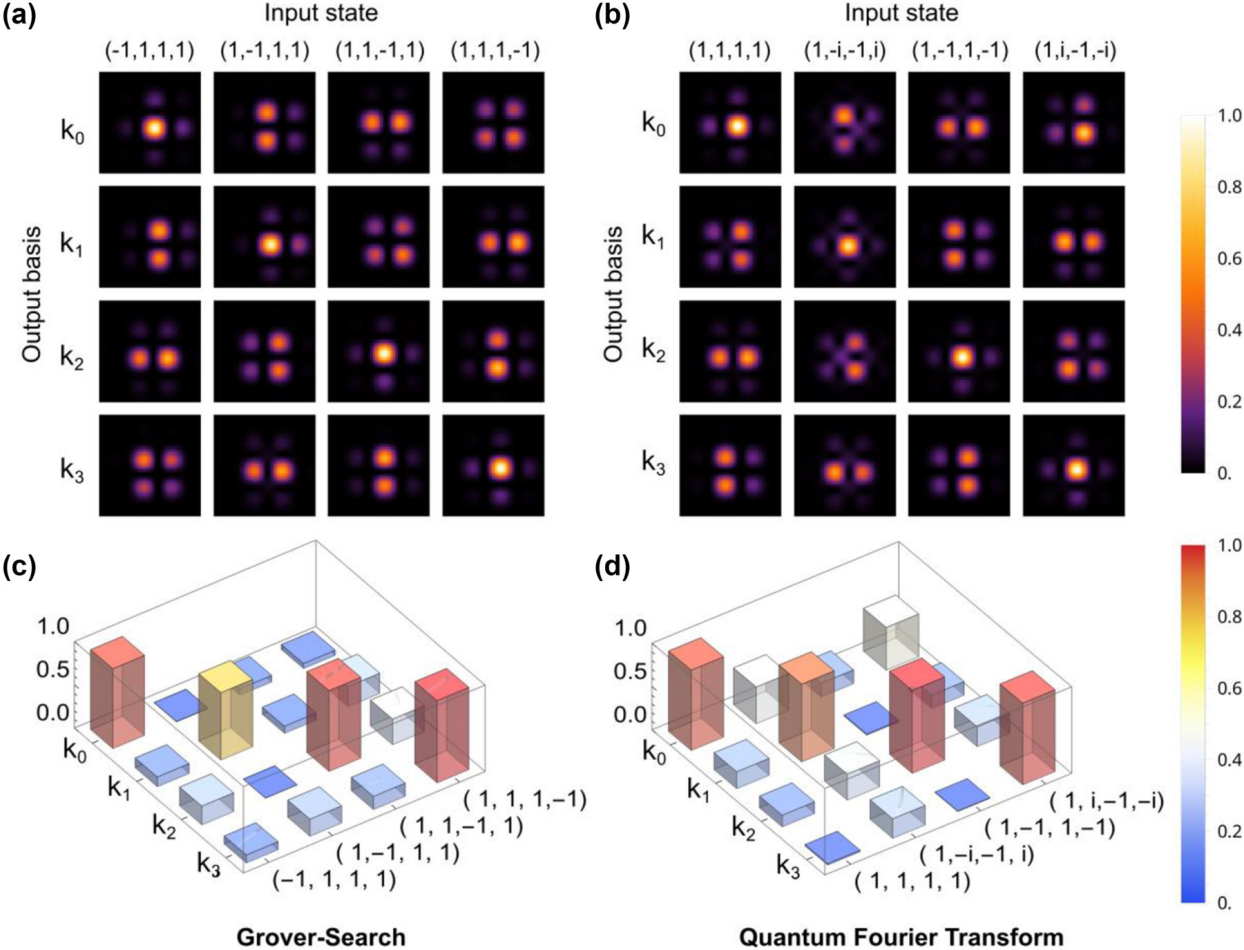
Experimental results using a quantum heralding photon source. (a and b) Interference patterns after up-sampling for the Grover’s search (GS) operation (left) and quantum Fourier transform (QFT) operation (right). Each column corresponds to a different input state, and each row shows the interference patterns formed in designated directions. (c and d) Normalized fitted output state amplitudes extracted from the interference patterns for the respective operations. The fitting method approximates the field amplitude at the center of the pattern by modeling the interference of the four metalenses.

On the other hand, using a quantum light source presents an additional challenge in extracting information due to the lower number of photons reaching the single-photon camera. To mitigate the impact of environmental noise in the situation of a smaller number of signal photons, we adopt the heralding technique (as shown in Figure 2(d)). In this case, we use a significantly smaller number of pixels on the camera, around 40 × 40 pixels, to capture the entire interference patterns. This allows us to ensure an adequate number of photons per pixel, enabling data acquisition within a manageable time duration. For the comparison of the results using classical laser and heralded single photons, please refer to the Supplementary Materials. In principle, for our implementation, it is sufficient to place four single-photon detectors to obtain the output state precisely at the four exit directions for a two-qubit operation. However, using a single-photon camera allows us to capture the full interference image. These additional detectors (now roughly around each exit direction) provide additional output bases, which are helpful for establishing a more robust measurement of the original output state by fitting the interference pattern, in a similar spirit to error correction. Here, we develop a fitting procedure for each interference pattern formed at a particular output direction, assuming that only four waves from the involved metalenses interfere with each other. The fitting procedure extracts the complex field amplitudes exiting each metalens. Based on this fitting procedure, we can effectively perform a 4× up-sampling of the interference pattern images, which are presented in Figure 4(a) and (b) for the GS and QFT, respectively. Further details regarding the up-sampling process from the raw images using the physical fitting model are provided in the Supplementary Materials. The up-sampled interference patterns are displayed in a tabular form with each column labeling the input state and each row labeling the output interference pattern at individual output directions (output basis), resembling the classical results shown in Figure 3 by reading out the individual interference patterns in a row-major manner. At the same time, we have also obtained the $\left|\psi_{i}^{\left(\text{out}\right)}\right|$ (continuous values now) at each output mode $\left|i\right\rangle$ during the fitting process. These values are presented in the same tabular form in Figure 4(c) and (d) for the GS and QFT, respectively. In both cases, a comparison is made to the expected results (identity matrix), resulting in a root-mean-square error (RMSE) of 0.148 for all 16 elements in the GS and 0.216 for the QFT. It is worth noting that the larger error observed at the **k**_0_ direction in QFT (as shown in Figure 4(d)) can be attributed to the presence of residual beams, which also appear in Figure 3(c), contributing to the larger RMSE in the QFT case. We note that we can scale-up the current approach by adding more lenses to the metalens array. Figure S3 in Supplementary Materials shows an example of four qubits operation with totally 16 lenses. While further scale-up relies much on how accurately the interference pattern can form from different lenses, we envision the current approach can be immediately useful to miniaturize part of a large quantum circuit. We also note that in our current sample, as the center lens is shared between two algorithms, there can be some output directions that are chosen not to read at decoding for a specific algorithm. So there is a dedicated balance between programmability and transmission efficiency, which can be further improved by optimizing the design matrix in minimizing the number of used output directions or adopting dielectric metasurfaces to increase the overall transmission efficiency.

## 3 Conclusions

In conclusion, we have developed a metalens array platform capable of implementing quantum algorithms with single photons. The functionality of the platform has been validated using both classical and quantum light sources. By selectively exciting subsets of metalenses and interpreting the interference patterns at specific output directions, we have demonstrated the programmability of quantum algorithms and operations, exemplified by the demonstration of Grover’s search (GS) and Quantum Fourier Transform (QFT) in this work. Such a platform holds promise for miniaturizing quantum optical circuits and algorithms, opening up possibilities for metasurface applications. By the demonstration using a single-photon quantum source, our scheme presents promising prospects for future works. Specifically, our scheme can be further extended to probe more information, such as about the metasurface operation by leveraging multiphoton quantum interference [29]. For example, by illuminating a pair of lenses using two photons and measuring the coincidence of any two output directions, we can probe out the phase difference between the competing process within the transfer matrix, sharing a similar principle as in boson sampling [7], [21]. While the scalability to a larger number of qubits through this approach requires further exploration, it already offers the opportunity to ‘factorize’ certain components within a larger-scale quantum computation. The metasurface can effectively reduce the size and eliminate the need for optical alignment in the factorized part. While we have demonstrated resource sharing between two operators on the same metasurface, further optimization of resource management can be explored in future developments. Additionally, the current work utilizes a geometric metasurface with robust operation, relying on metallic components rather than local resonances. However, it may be advantageous to adopt dielectric metasurfaces in the foreseeable future to achieve higher transmission efficiency, thereby enabling the use of larger metalens arrays for quantum operations.

## 4 Methods

### 4.1 Sample fabrication

Prior to fabrication, the glass substrate is immersed in concentrated sulfuric acid for 24 h and then cleaned successively with distilled water and acetone. A 50 nm-thick silver film is then deposited onto the substrate by an E-beam Evaporator (AST 600 EI Evaporator), operating at a deposition rate of $1\overset{{}^{\circ}}{A}/\text{s}$ . Finally, a focus ion beam (FEI Helios G4 UX, 30 kV, 41 pA) is used to write the designed nanoslot pattern on the prepared silver film.

### 4.2 Experimental and measurement details

The full experimental setup is presented in the Supplementary Materials. We use a 2-mm-thick type-II BBO and a 200 mW 405 nm laser (CrystaLaser DL-405-400) to generate the polarization-entangled photon pairs in a state of 1/ $\sqrt{2}$ (|HV⟩ + |VH⟩). The photons are split into the signal arm and heralding arm using a prism. To compensate for the translational and longitudinal walk-off effects of the photon pairs, a half-wave plate (HWP), with an optical axis at 45° with the horizontal axis, and a BBO with half of the thickness of the main BBO are installed in both arms. On the heralding arm, a polarizer selectively filters horizontally (H) polarized photons, such that the heralding process only triggers imaging for those photons with vertical (V) polarization. The heralding photons are then detected using a single photon counting module (SPCM) (Excelitas-SPCM-800-14-FC), which triggers the Single Photon Avalanche Diode (SPAD) camera (Pi Imaging SPAD 512S). On the signal arm, the photons are then coupled into a 10 m single-mode fiber to the imaging setup. Since the propagation in the fiber alters the polarization of the signal photons, a QWP and HWP are used for polarization correction. The orientations of these wave plates are calibrated by using a correlator (UQD-Logic-16) to obtain the maximum coincidence between the signal arm (SPCM1) and heralding arm (SPCM2 in the flip mirror path). Alternatively, we can replace the input of the signal fiber with the light from 810 nm laser (OBIS LX 808 nm 150 mW Laser), polarized with a V-polarizer as our classical source.

The SLM (Holoeye Pluto 2.1), only responds to V-polarized photons, generates the input state and selectively excites the metalens. To control the amplitude from the phase-SLM, some light is diverted and blocked by an iris positioned at the focal plane of a lens with *f*_1_ = 40 cm. This lens and a 10× objective are used to image the SLM plane to the metasurface plane. The first QWP, with a fast axis oriented at the diagonal, converts V-polarized light to LCP. Upon interacting with the metasurface, the photons diffract into different output directions. The second QWP, with a fast axis oriented at the anti-diagonal, and an H-polarizer are used to convert the polarization back to linear polarization that is orthogonal to the input light in the signal arm. A 20× objective (before the second QWP) is used to map the focal plane of the metasurface to the camera. The interference patterns corresponding to different output directions are formed at the focal plane of the metalens array and captured by the SPAD camera.

For our classical result, the same camera is used in the photon counting mode with an exposure time of 5 ms. The image size for the classical result is 195 × 195 pixels and is directly displayed in Figure 3. For our heralding result, a 0.2× magnification system is added to shrink the image size down to 39 × 39 pixels. The heralded images are all retrieved using 20,000 frames with external triggers from SPCM (16 ns coincidence window) and with background white noise subtracted. The background is measured using the same triggering setting with blocked signal photons. Each time frame spans 300 ms with a maximum of 255 photon counts in each pixel. These images are split into nine 13 × 13 pixels, which are used for fitting (details in the Supplementary Materials), and the results are displayed in Figure 4.

## Supplementary Material

Supplementary Material Details
