# Supplementary material for: Metasurface for programmable quantum algorithms with classical and quantum light
Source: Nanophotonics. 2024 Feb 21;13(6):927–36. doi: 10.1515/nanoph-2023-0844 (PMC11614333; doi:10.1515/nanoph-2023-0844)
Supplement: Supplementary file 1 — Supplementary Material Details [file j_nanoph-2023-0844_suppl_001.pdf]

Metasurface for programmable quantum algorithms with classical and quantum light

Randy Stefan Tanuwijaya[1], Hong Liang[1], Jiawei Xi[1], Wai Chun Wong[1],
Tsz Kit Yung[1], Wing Yim Tam[1], and Jensen Li[1*]
[1]Department of Physics, The Hong Kong University of Science and Technology,
Clear Water Bay, Kowloon, Hong Kong, 999077, P. R. China

Supplementary Information

Supplementary Sections

Supplementary 1 Metasurface Profile 2
Supplementary 2 Experimental Setup 7
Supplementary 3 Fitting Method 9

Supplementary Figures

Supplementary Figure S1 Power distribution of each metalens 3
Supplementary Figure S2 Simulation results with more embedded operations. 4
Supplementary Figure S3 Simulation results of 4 qubits QFT 6
Supplementary Figure S4 Full experimental setup 7
Supplementary Figure S5 Library of interference patterns 10
Supplementary Figure S6 Comparison of results using a classical light source and heralding photon source 11

Supplementary 1 Metasurface Profile

To encode multiple quantum operations into a single metasurface, it is necessary to design a global U-matrix or a transfer matrix. This matrix specifies the amplitude and phase modulation, denoted as U_{ij}, for each input mode $|j\rangle$ to each output mode $|i\rangle$. In the case of our metalens array, we have designed a 9×9 transfer matrix, where each column corresponds to a specific metalens, and each row corresponding to a specific exit direction (ordered in a row-major manner). In this context, it is worth noting that the transfer matrix is not limited to being unitary. For the sample utilized in our experiment, the design transfer matrix is given as follows:

$$U = \begin{pmatrix} -1 & 1 & 0 & 1 & 1 & 0 & 0 & 0 & 0 \\ 1 & -1 & 0 & 1 & 1 & 0 & 0 & 0 & 0 \\ 0 & 0 & 0 & 0 & 0 & 0 & 0 & 0 & 0 \\ 1 & 1 & 0 & -1 & 1 & 0 & 0 & 0 & 0 \\ 1 & 1 & 0 & 1 & -1 & -1 & 0 & -1 & -1 \\ 0 & 0 & 0 & 0 & -1 & -\mathrm{i} & 0 & 1 & \mathrm{i} \\ 0 & 0 & 0 & 0 & 0 & 0 & 0 & 0 & 0 \\ 0 & 0 & 0 & 0 & -1 & 1 & 0 & -1 & 1 \\ 0 & 0 & 0 & 0 & -1 & \mathrm{i} & 0 & 1 & -\mathrm{i} \end{pmatrix} \tag{S1}$$

By selecting the metalens excitation and the projected interference pattern, we can select any arbitrary submatrix of the 9×9 transfer matrix as our programmable quantum operation. For example, the submatrix of the transfer matrix formed by the 1st, 2nd, 4th, and 5th rows and columns is $\hat{U}_{\mathrm{GS}}$, while the submatrix formed by the 5th, 6th, 8th, and 9th rows and columns is $\hat{U}_{\mathrm{QFT}}$ with an added π global phase. Notably, the 3rd and 7th columns, corresponding to the upper-right and lower-right lenses, are composed of all zero values. These two lenses are designed using the same methodology as the other lenses in the fabrication (Fig. 2a), such that they consist of orthogonal slots that generate zero cross-circular polarization.

To design the metasurface transmission profile based on a given transfer matrix, we used the following equation, which directly integrates a quadratic focusing phase profile.

$$t_j(\mathbf{r}) = \frac{1}{\max\limits_{j,r}(t_j(\boldsymbol{r}))} \exp\left(-\mathrm{i}\,\frac{2\pi}{\lambda}\sqrt{\left|\mathbf{r} + \mathbf{R}_j\right|^2 + f^2}\right) \sum_{i=0}^{8} U_{ij} \exp\left(\mathrm{i}\,\mathbf{k}_i \cdot \left(\mathbf{r} + \mathbf{R}_j\right)\right) \tag{S2}$$

where $\mathbf{r} = \boldsymbol{\rho} - \mathbf{R}_j$ is the position of the unit cell in the local coordinate for each lens ($\boldsymbol{\rho}$ corresponds position in to the global coordinate), $\mathbf{R}_j = 32p\,\left(n_{jx}\,\hat{\mathbf{x}}, n_{jy}\,\hat{\mathbf{y}}\right)$ is the origin of lens j, $\mathbf{k}_i = \frac{2\pi}{\lambda}\left(\sin\theta_{ix}\,\hat{\mathbf{x}}, \sin\theta_{iy}\,\hat{\mathbf{y}}\right)$ is the target direction wave vector, $f = 150\mu\mathrm{m}$ is the focal

length of the metalens, $p = 620\text{nm}$ is the periodicity of the unit cell, and $\lambda = 810\text{nm}$ is the operational wavelength of the metasurface. Here, $n_{jx}, n_{jy} \in \{-1, 0, 1\}$ are the position of the nine lenses relative to the center lens, and $\theta_{ix}, \theta_{iy} \in \{-10°, 0°, 10°\}$ are the target diffraction angle. Afterwards, we normalize the amplitude by the global maximum of all lenses, i.e., $\max_{j,\boldsymbol{\rho}}(t_j(\boldsymbol{\rho}))$ for all j and all sampled $\boldsymbol{\rho}$, to confine the transmission amplitude to the range that can be realized.

In our design methodology, each metalens can diffract light into different number of target directions. For instance, in the transfer matrix described in Eq. (S1), the center metalens diffracts light into seven directions, while the other metalenses diffract light into either zero or four directions. The principle to guarantee the power distribution according to $|U_{ij}|^2$ stems from both the amplitude and phase control of the individual unit cells. In the actual sample, this amplitude and phase control is achieved by controlling the angle of the nanoslot pairs (θ_1 and θ_2 in Fig. 2c) in the 2-by-2 nanoslots unit cell. Fig. S1a illustrates the phase profile to illuminate one lens at a time (with 100% input power over the circular area covering one metalens), and Fig. S1b illustrates the simulated power distribution of each metalens to a particular direction, in which each direction receives ~3.3% of the input power, with the center metalens achieving the highest design efficiency of ~23%.

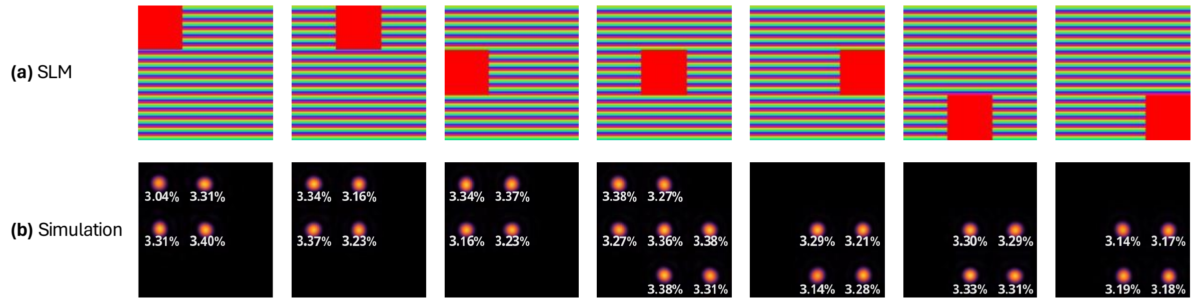

Fig. S1 | Power distribution of each metalens. a, SLM phase profile for illuminating only one lens at a time with 100% power. Red squares indicate which lens is currently illuminated. **b,** Numerical simulation of the diffraction pattern at the focal plane of the metalens. The percentage indicates the intensity distributed to a particular area.

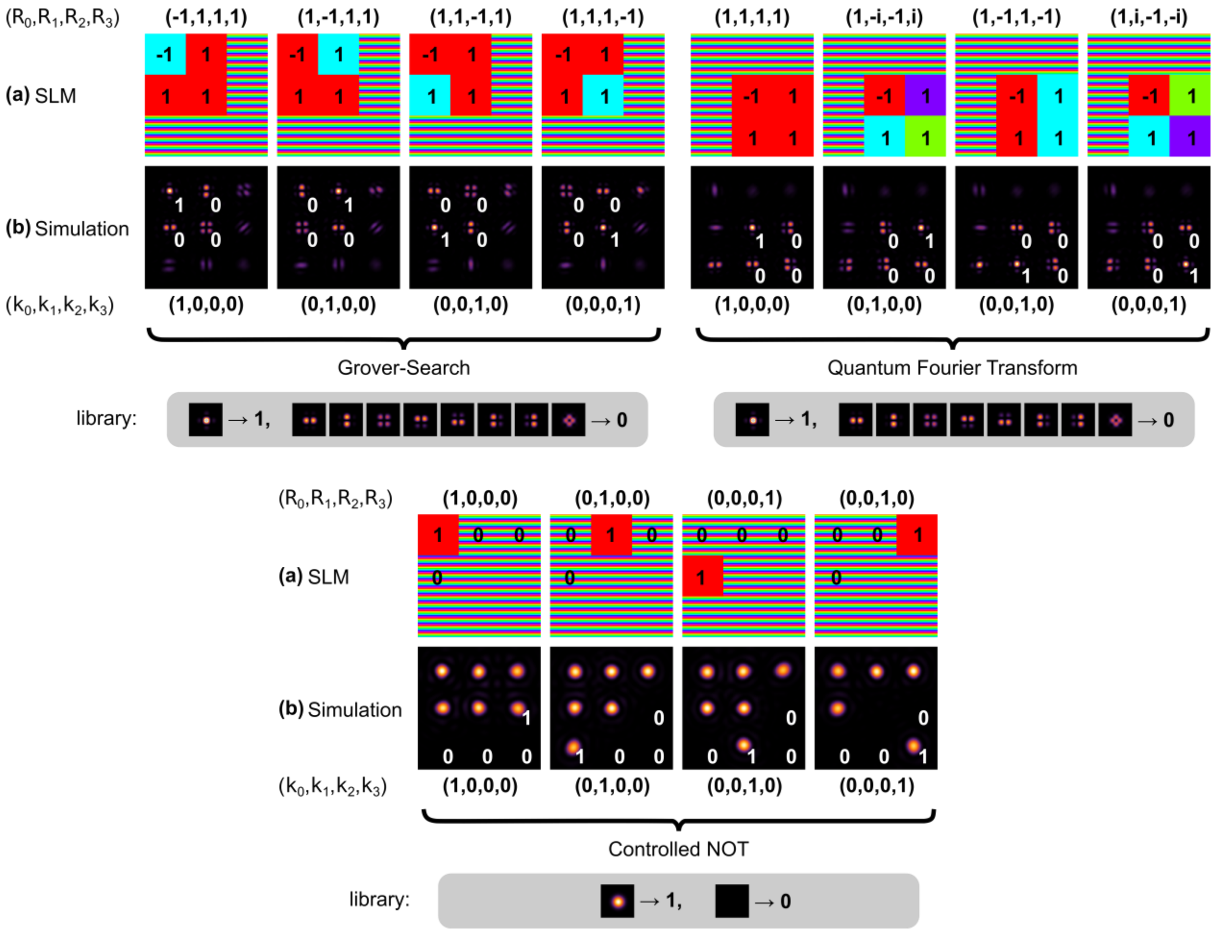

Fig. S2 | Simulation results with more embedded operations. a, SLM phase profile for each input state for the three operations (GS, QFT, and CNOT): four input states for each operation listed at the top of each column. Color code: red for 1, cyan for -1, green for i, and purple for −i, shaded regions (phase gradient) are 0 or inactive. **b,** Numerical simulation of the far-field interference pattern. The input and output states are labeled at the top and bottom of each column. The library for decoding the interference pattern into the output state for each operation is listed in the bottom inset.

For the entire (9 lenses) × (9 directions) matrix, there are unused matrix elements where we can embed more operations. For example, in addition to the GS and QFT operation, we designed global U'-matrix which encodes a CNOT operation well, which can be activated by selecting the submatrix spanned by 1st, 2nd, 3rd, and 4th columns with 6th, 7th, 8th, and 9th rows. For reference, the given U’-matrix is provided as follows:

$$U' = \begin{pmatrix} -1 & 1 & 1 & 1 & 1 & 1 & 0 & 0 & 0 \\ 1 & -1 & 1 & 1 & 1 & 0 & 1 & 0 & 0 \\ 1 & 1 & -1 & 1 & 0 & 0 & 0 & 0 & 1 \\ 1 & 1 & 1 & -1 & 1 & 0 & 0 & 1 & 0 \\ 1 & 1 & 0 & 1 & -1 & -1 & -1 & -1 & -1 \\ 1 & 0 & 0 & 0 & -1 & -\mathrm{i} & 1 & 1 & \mathrm{i} \\ 0 & 1 & 0 & 0 & -1 & 1 & -1 & -1 & 1 \\ 0 & 0 & 0 & 1 & -1 & 1 & -1 & -1 & 1 \\ 0 & 0 & 1 & 0 & -1 & \mathrm{i} & 1 & 1 & -\mathrm{i} \end{pmatrix} \tag{S3}$$

Fig. 1b and Fig. S2 shows the metasurface transmission profile and the far-field simulation result of the profile designed using U'-matrix. It is important to note that despite the presence of interference patterns beyond the four specified exit directions (unlabeled patterns corresponding to rows outside the selected submatrix), these additional patterns do not impact our results and can be disregarded when extracting the desired output state. Furthermore, it is worth emphasizing that different lens configurations require distinct libraries for interpreting the interference patterns, exemplified by the two different libraries used in GS/QFT and CNOT. For instance, in the cases of GS and QFT operations, the interference patterns are formed by four lenses arranged in a rectangular configuration, utilizing the same library for both operations. On the other hand, the CNOT operation does not involve interference among multiple lenses, and the resulting output state corresponds to a single Gaussian mode of the selected lens. For a more detailed discussion on the modes and interference patterns, please refer to Supplementary 3.

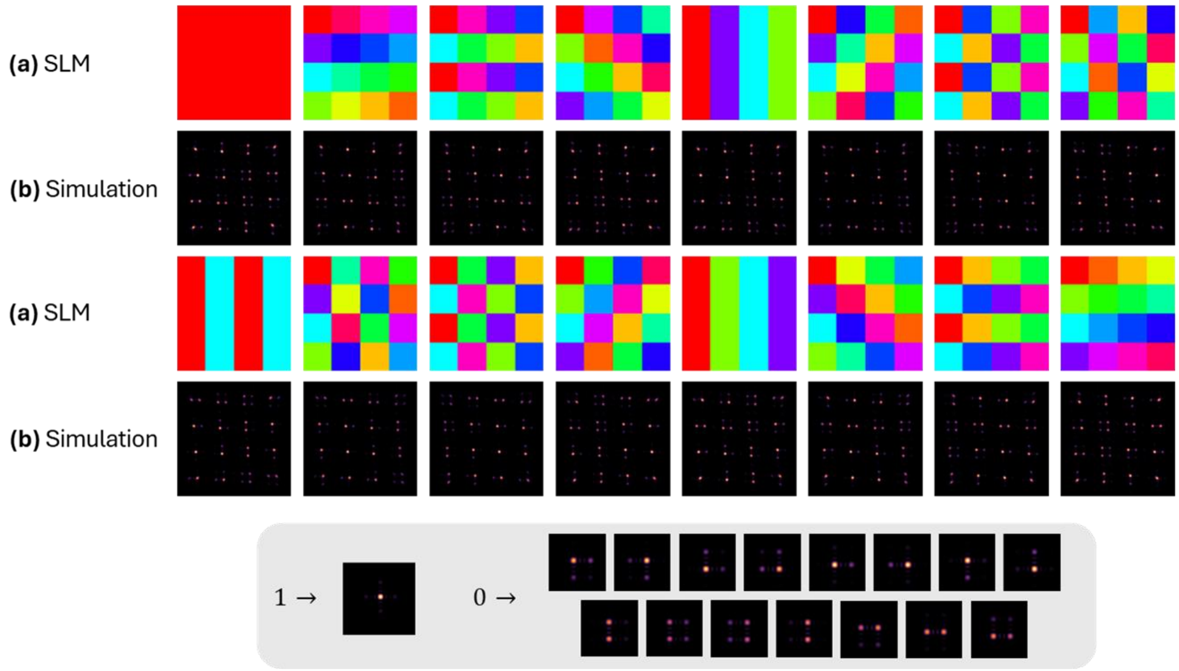

Fig. S3 | Simulation results of 4 qubits QFT. a, SLM input phase profile as 4-qubit input state. **b,** Far-field interference pattern simulation result, forming 4-by-4 interference pattern array corresponding to the 4-qubit output state. The inset at the bottom shows the pattern library in decoding output state.

Figure S3 illustrates the same design principle (Eq. S3) used to realize 4-qubits QFT operation. In this case, 4-by-4 metalenses are used to realize the 4-qubits (16) input states, with each of them composed of 40 unit-cells (with periodicity $p = 620\text{nm}$). Each metalens is designed to diffract light into also 4-by-4 target directions, with separation of 6° in angle between neighbouring directions. Fig. S3a shows the SLM input phase profiles of different spatial frequencies, and Fig. S3b shows the corresponding simulation results of the interference patterns at the focal plane ($f = 500\mu\text{m}$). The results show value one can be successfully decoded at the correct output direction corresponding to the spatial frequency of SLM input (decoding library shown in the lower panel).

Supplementary 2 Experimental Setup

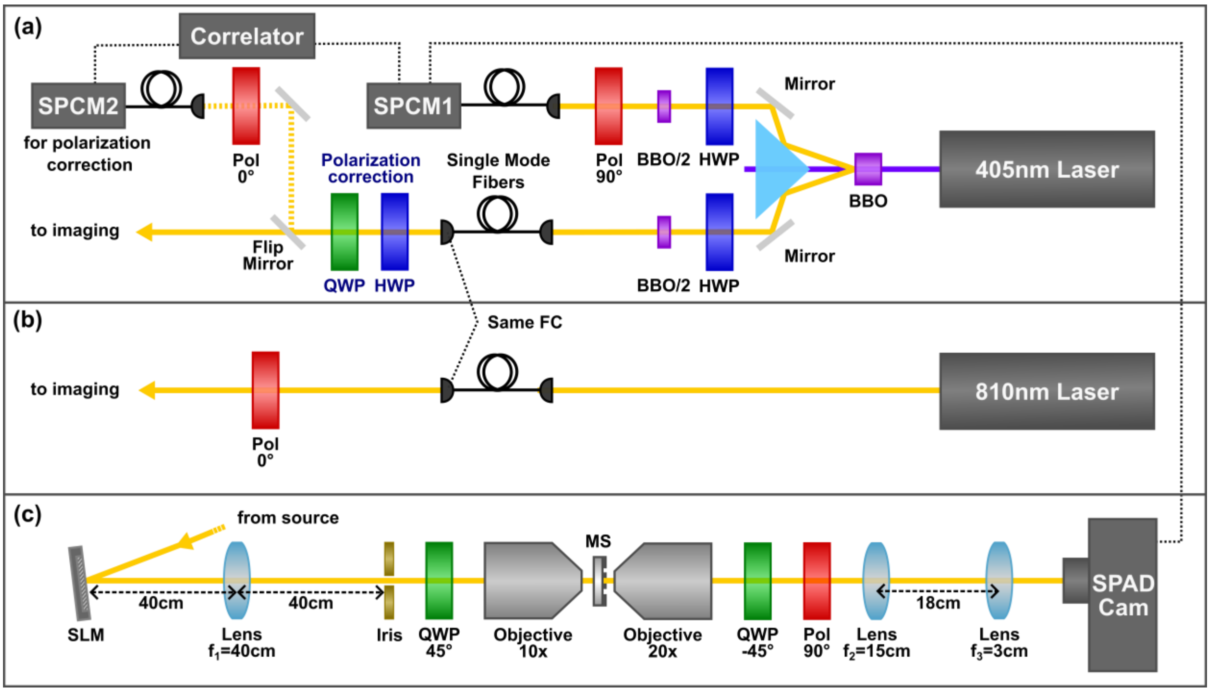

Fig. S4 | Full experimental setup. a, Entangled photon source generation by pumping 405nm laser to type-II β-barium borate (BBO) **b,** Classical source using 810nm laser. **c,** imaging setup, a single photon camera imaging the focal plane of the metalens array.

For our quantum source generation in Fig. S4a, we use a 2-mm-thick type-II BBO and a 200mW 405 nm laser (CrystaLaser DL-405-400) to generate the polarization-entangled photon pairs in a state of $1/\sqrt{2}(|HV\rangle + |VH\rangle)$. The half-opening angle of the generated photon pairs is designed to be 3°. The photons are split into the signal arm and heralding arm using a prism. To compensate for the translational and longitudinal walk-off effects of the photon pairs, a half-wave plate (HWP), with an optical axis at 45° with the horizontal axis, and a BBO with half of the thickness of the main BBO are installed in both arms.

On the heralding arm, a polarizer selectively filters horizontally (H) polarized photons, such that the heralding process only triggers imaging for those photons with vertical (V) polarization. The heralding photons are then detected using a single photon counting module (SPCM) (Excelitas-SPCM-800-14-FC), which triggers the Single Photon Avalanche Diode (SPAD) camera (Pi Imaging SPAD 512S). Each trigger from the SPCM would turn on the camera for a detection window of 16ns. On the signal arm, the photons are then coupled into a 10m single-mode fiber to the imaging setup. Since the propagation in the fiber alters the polarization of the signal photons, a QWP and HWP are used for polarization correction. The orientations of these wave plates are calibrated by using a correlator (UQD-Logic-16) to obtain

the maximum coincidence between the signal arm (SPCM1) and heralding arm (SPCM2 in the flip mirror path). Alternatively, we can replace the input of the 10m single-mode fiber with the 810nm laser (OBIS LX 808 nm 150 mW Laser), along with a vertical polarizer as our classical source, as illustrated in Fig. S4b.

Fig. S4c illustrates the setup for our imaging system. The SLM (Holoeye Pluto 2.1) only responds to V-polarized photons, generates the input state, and selectively excites the metalens. To control the amplitude from the phase-SLM, some light is diverted and blocked by an iris positioned at the focal plane of a lens with $f_1 = 40\text{cm}$. This lens and a 10x objective are used to image the SLM plane to the metasurface plane. The first QWP, with a fast axis oriented at the diagonal, converts V-polarized light to LCP. Upon interacting with the metasurface, the photons diffract into different output directions. The second QWP, with a fast axis oriented at the anti-diagonal, and an H-polarizer are used to convert the polarization back to linear polarization that is orthogonal to the input light in the signal arm. A 20x objective (before the second QWP) is used to map the focal plane of the metasurface to the camera. A 0.2x magnification system ($f_2 = 15\text{cm}$ and $f_3 = 3\text{cm}$) is used to shrink the image size for imaging with a quantum source. Lastly, the interference patterns corresponding to different output directions are formed at the focal plane of the metalens array and captured by the SPAD camera.

Supplementary 3 Fitting Method

In the main text, we demonstrate that the output state can be extracted continuously, based on a fitting procedure to extract the features of the interference pattern. To construct our fitting procedure, we first consider the field one metalens will project to one of its focal spots. By utilizing Fourier method, this field can be approximated as a Gaussian profile on around the focal spot, with a phase gradient proportional to its position relative to the optical axis. Thus, the field profile around the exit direction $\mathbf{k}_i$ due to lens $\mathbf{R}_j$ is given by:

$$E_{ij}(\mathbf{k};\{\boldsymbol{k_i},s,\sigma\}) = e^{-\frac{s^2|\mathbf{k}-\mathbf{k}_i|^2\sigma^2}{2}} e^{-\mathrm{i}\,s\,(\mathbf{k}-\mathbf{k}_i)\cdot\mathbf{R}_j} \tag{S4}$$

where σ is a fitting parameter proportional to the effective radius of the metalens. Notably, now the k-space can be regarded as the camera plane, such that s is a fitting parameter related to the magnification of the imaging system.

Around each direction, we assume the fields from each lens to interfere with the other lenses with different amplitude and phase $A_{ij}e^{\mathrm{i}\phi_{ij}} = U_{ij}\psi_j^{(in)}$. Adding the fields from each involved metalens (with a scaling factor a and noise term b), the fitting function to model the intensity profile of the interference pattern is given by:

$$I_i(\mathbf{k};\{\boldsymbol{k_i},s,\sigma,a,b,,A_{i0}\ldots A_{i3},\phi_{i0}\ldots\phi_{i3}) = a\left|\sum_{j=0\ldots3} E_{ij}(\boldsymbol{k};\{\boldsymbol{k_i},s,\sigma\})\,A_{ij}e^{\mathrm{i}\phi_{ij}}\right|^2 + b, \tag{S5}$$

To build our library for pattern matching, we browsed through all possible interference patterns formed by four metalenses in a rectangular configuration, i.e., $\mathbf{R}_j = 32p\,\{(0\hat{\mathbf{x}},0\hat{\mathbf{y}}),\ (0\hat{\mathbf{x}},1\hat{\mathbf{y}}),\ (1\hat{\mathbf{x}},0\hat{\mathbf{y}}),\ (1\hat{\mathbf{x}},1\hat{\mathbf{y}})\}$ (note that the global shift of $\mathbf{R}_j$ does not affect the interference profile). Furthermore, we constrained all the fitting amplitude A_{ij} to be one, and four phases after the metalens ϕ_{i1} to ϕ_{i4} to be an integer multiple of $\pi/2$. By testing all combinations of ϕ_{i1} to ϕ_{i4} we obtained our intensity profiles library corresponding to each output state amplitude, which is shown in Fig. S5. Notably, some amplitudes are highly degenerate, in which the same output state a amplitude can be represented by different intensity profiles. The fully destructive and constructive interference patterns, corresponding to $\psi_i^{(\mathrm{out})}$ equal to zero and one, are shown as an inset of Fig. 3 in the main text.

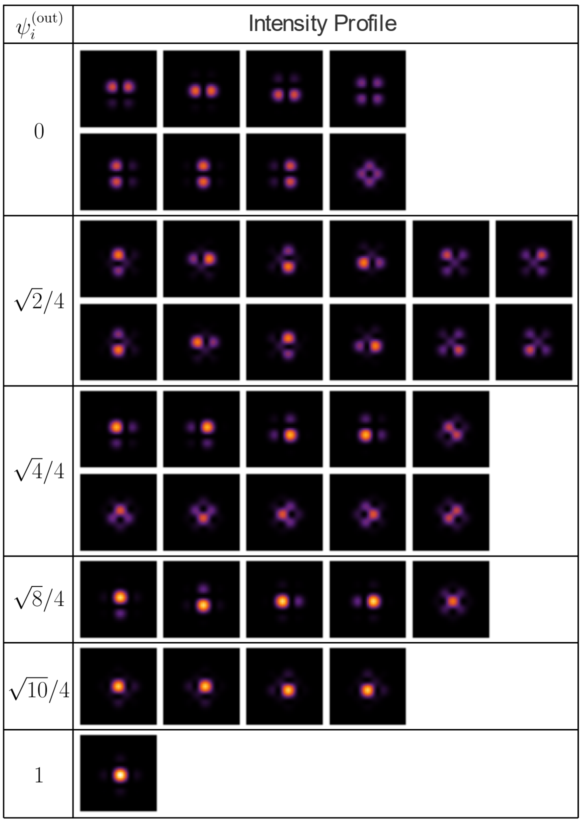

Fig. S5 | Library of interference patterns. All possible interference patterns from four rectangular lenses (sorted and grouped by the output state amplitude, with a constraint of $A_{ij} = 1$ and $\phi_{ij} = n\pi/2$, where n is integer)

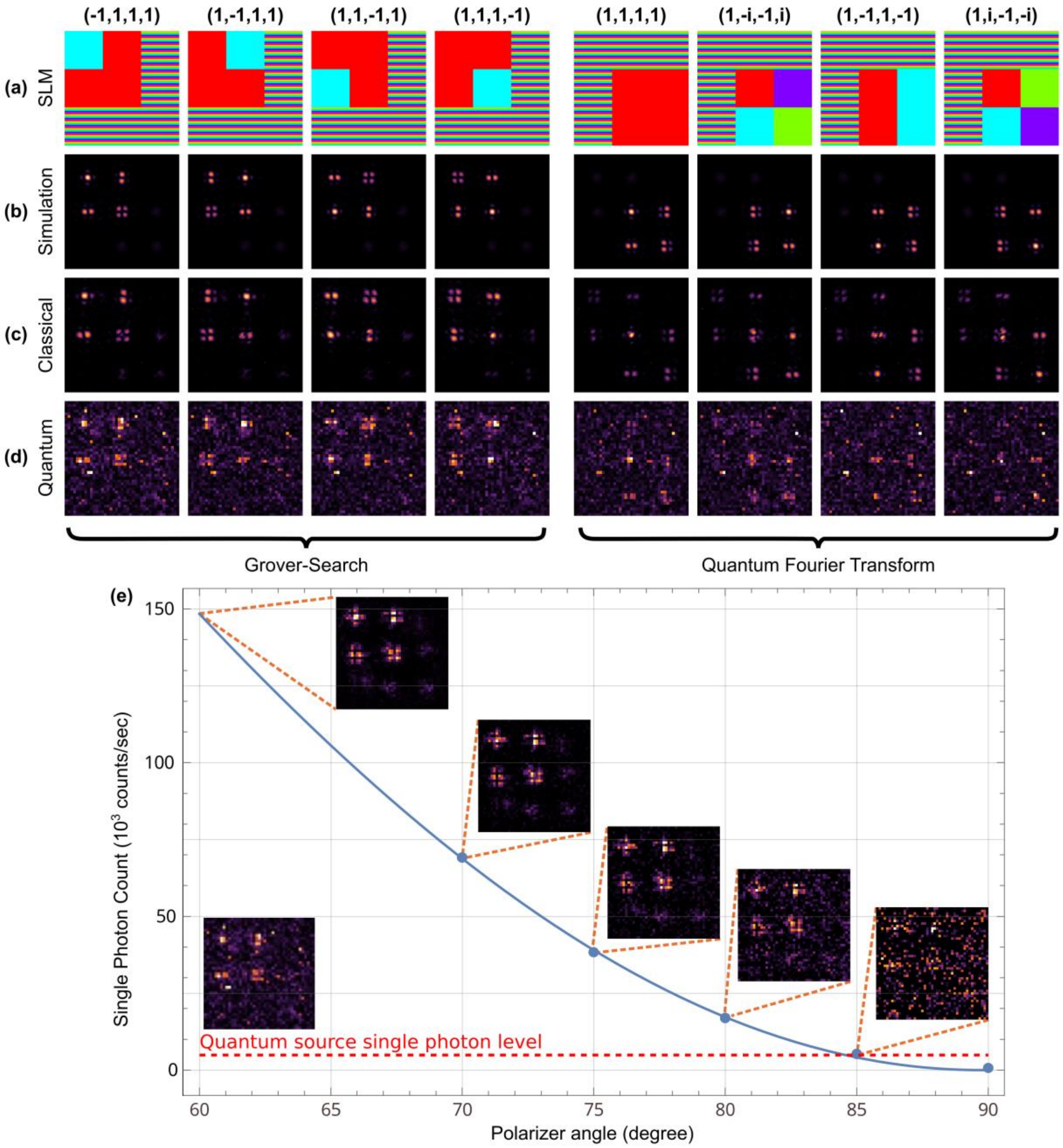

Fig. S6 | Comparison of results using a classical light source and heralding photon source. a, Input state encoded into SLM phase profile. **b,** Simulation result of the farfield interference pattern. **c,** Experimental result using an 810nm laser source, captured using an SPAD camera in photon counting mode. **d,** Experimental result using a heralding photon source, captured using SPAD camera in gated mode (white noise subtracted). **e,** Comparison of heralded single photon coincidence image versus attenuated classical laser intensity image at similar power level. The images along the curve show the background-subtracted intensity image by varying the input power level of the classical laser using ND filters with a polarizer as tuning. The red-dashed line shows the single-photon power level of the quantum source. The image on the lower-left corner shows the background-subtracted coincidence image of heralded single-photon source.

Fig. S6 shows the quality comparison of the experimental results obtained by using the classical and the quantum light source. Fig. S4(a-c) are the same as Fig. 3(a-c), while Fig. S6d shows 39x39 pixels of white noise subtracted coincidence images when using the quantum light source. Fig. S6e shows the measured input single photon count level for attenuated classical light using multiple ND filters (down to ~600k single photon counts per second) and varying polarizer angle (blue points and curve for the measured and interpolated counts) and the red dashed line marks the single photon count for our quantum source (~5000 single photon counts per second). In particular, the quantum source single photon power level is equivalent to the classical laser attenuated with ND filters and 85° polarizer angle (~5200 single photon counts per second). The insets along the blue curve shows the far-field interference image obtained at different classical laser power level by varying the polarizer angle. The inset on the lower left shows the far-field interference coincidence image obtained by using heralded single photon. Comparing the classical laser result at the same power level with the heralded single photon source (attenuated with ND filters and polarizer with 85° angle), it shows that heralding technique is necessary to obtain acceptable SNR for single photon source [1, 2].

To utilize these coincidence images for fitting, we first separate each interference pattern into 13x13 pixels images as the input data for our fitting procedure. Here, we use a small number of pixels to focus more signal photons into the same camera pixels. Our experimental result using the heralding photon source yields a low number of coincidence counts, which is roughly 10000 signal coincidence counts for 100 minutes of exposure time (20000 white noise subtracted frames, 300ms frame time each). Based on these coincidence image, we first overlap the images of different inputs and fit the center positions for each target directions to obtain the fitting parameter $\boldsymbol{k}_i$ for $i = 0 \ldots 3$. Then, we performed a nonlinear fitting to simultaneously optimize the fitting the parameters $s, \sigma, a,$ and b along with the phases after the four lenses ϕ_{i1} to ϕ_{i4} for all the interference patterns (assuming $A_{ij} = 1$). Specifically, the fitting parameters are $s, \sigma, a,$ and b are shared across all the fitted interference patterns, while the fitting parameter $\boldsymbol{k}_i = \{k_i^x, k_i^y\}$ are shared across the interference pattern with the same direction. Based on the fitted phases, the fitted interference patterns $I_i(\boldsymbol{k}; \{\boldsymbol{k_i}, s, \sigma, a, b, 1 \ldots 1, \phi_{i0} \ldots \phi_{i3}\})$ are shown in Fig. 4a and 4b (with 4x up-sampling) and the output field amplitudes at the center of the interference pattern $\psi_i^{(\mathrm{out})}$ (Eq. S6) are shown in Fig. 4c and 4d.

$$\psi_i^{(\mathrm{out})} = \frac{1}{4}\sqrt{\frac{1}{a}\left(I_i(\mathbf{k_i}; \{\boldsymbol{k_i}, s, \sigma, a, b, 1 \ldots 1, \phi_{i0} \ldots \phi_{i3}\}) - b\right)} = \frac{1}{4}\left|\sum_{j=0\ldots3} e^{\mathrm{i}\phi_{ij}}\right| \qquad (S6)$$

1. Kim, J. et al. Heralded single-pixel imaging with high loss-resistance and noise-robustness. *Applied Physics Letters* **119**, 244002 (2021).
2. Zhou, J. et al. Metasurface enabled quantum edge detection. *Sci. Adv.* **6**, eabc4385 (2020).
